# Changes in mental health during participation in Art Pharmacy: a longitudinal study of a U.S. arts-based social prescribing program

**DOI:** 10.3389/fpubh.2026.1799519

**Published:** 2026-04-30

**Authors:** Lucy Rabinowitz Bailey, Peter R. DiMilia

**Affiliations:** 1 Department of Mental Health, Johns Hopkins University, Bloomberg School of Public Health, Baltimore, MD, United States; 2 Art Pharmacy, SocialRx Inc, Atlanta, GA, United States

**Keywords:** arts prescribing, general estimating equations, mental health, program evaluation, social prescribing

## Abstract

**Introduction:**

Loneliness and social isolation are increasingly recognized as public health challenges linked to higher health care costs and poor health outcomes. As mental health needs continue to outpace the availability of behavioral health resources, there is growing need for cost-effective, widely accessible, whole-person approaches, such as social prescribing.

**Methods:**

This study examines changes in self-reported mental health during participation in Art Pharmacy, a US-based social prescribing program, among adults referred through health care and community partners. We analyzed changes in mental health over time using a quasi-experimental single-group longitudinal design. The primary outcome was mental health and psychological wellbeing measured with the WHO-5 Wellbeing Index. Generalized estimating equations examined changes across follow-ups.

**Results:**

WHO-5 scores increased significantly over time. In baseline-adjusted analysis (*n* = 239), WHO-5 scores increased at all follow-ups (*p* < 0.001), with peak mean increases of 18–20 points at follow-ups 6–8 and sustained through follow-up 9. Increases exceeded the 10-point threshold for meaningful change on the WHO-5. Baseline WHO-5 scores predicted follow-up levels and participants showed similar improvement trajectories. Similar patterns were observed in demographic-adjusted sensitivity analysis (*n* = 145). Age, race, and ethnicity were not significantly associated with follow-up wellbeing scores, and treatment duration did not show additional associations beyond program progression.

**Discussion:**

Findings suggest that participation in an arts-based social prescribing program was associated with improvements in mental health over time. The results contribute preliminary evidence of an association between participation in arts-based social prescribing and health, and support the need for further evaluation of social prescribing in controlled studies.

## 1 Introduction

Attention has grown on the epidemic of loneliness and social isolation as public health concerns, given their associations with poor health outcomes, higher health care costs, and increased mental health concerns. Mental health concerns are inextricably linked to physical health outcomes, as seen through associations between depression and lower medication adherence in chronic disease management, and between behavioral health treatment and cancer outcomes (1–6). Research has demonstrated that lack of social connection contributes to adverse health effects comparable to smoking 15 cigarettes daily (7). There is an established reciprocal relationship between loneliness and depression, underscoring the need for upstream approaches that address both concerns simultaneously. Despite these well-documented interconnections, mental health needs among the most vulnerable populations, including older adults and young adults, continue to outpace the availability of behavioral health resources (8).

In 2020, 24% of older adults in the US were socially isolated and 18% reported depression symptoms. Among youth and young adults, suicidal ideation increased by 36% between 2009 and 2019, and psychiatric emergency department visits increased by 28% between 2011 and 2015 (9). These concerns have increased since COVID-19 pandemic, reflecting the cumulative effects of lockdowns, isolation, grief and loss, and other widespread stressors experienced across generations (10). Further, increased social media use and screen time has led to additional strain on mental health (11–13). In 2024, 23% of adults in the US experienced a mental illness, and 2022 marked the highest number of suicide deaths recorded nationally (14).

Despite the growing burden of mental health needs, many adults in the US face challenges in accessing holistic health supports. These challenges include behavioral health workforce shortages (15), stigma for seeking mental health care (16), lack of insurance or underinsurance (17), insufficient coverage (18), and the high cost of mental health services (19). These challenges exacerbate existing health disparities by socioeconomic status, racial and ethnic background (20, 21), language, and geographic residence (22). Collectively, this highlights the need for spaces that foster social connection and support the development of healthy relationships to mitigate the risk for developing debilitating health concerns.

### 1.1 Addressing unmet health needs with social prescribing

Supporting health across the lifespan requires whole person approaches that address mental and social aspects of wellbeing, alongside clinical treatment. Social prescribing offers a promising strategy to address these concerns through equitable, cost-effective opportunities for promoting health and wellbeing (23–26). Social prescribing enables health care providers to deliver whole person care (27) by referring patients to non-clinical community-based activities aligned with their interests, needs, and health goals (28–32). While these models are becoming widely utilized in countries with national health systems [like the United Kingdom and Canada (33, 34)], there is growing momentum in the US to integrate social prescribing into health service delivery to enhance health and wellbeing while addressing loneliness and isolation that drives behavioral and physical health concerns (30, 35–39).

In social prescribing, health care providers (or any care team member) refer patients to targeted non-clinical community supports, such as arts and cultural activities, volunteering, group exercise, or gardening (40, 41). Providers identify mental health or social connection needs (e.g., depression or social isolation screening) and issue a “social prescription,” which includes a referral to a link worker (42, 43). The link worker and patient then co-develop a plan for engagement in community activities, with ongoing patient engagement, support, and monitoring of health and social outcomes to identify additional needs over time (40).

Social prescribing was originally implemented among older adults and then expanded to youth and individuals with complex care needs (32, 35, 44–47). These models have demonstrated effectiveness at improving wellbeing (48); producing lifestyle changes which boost healthy behaviors such as increased physical activity (49); reducing loneliness (44), depression and anxiety (50, 51); and enhancing patient-provider relationships (52, 53). Further, social prescribing programs have demonstrated positive social returns on investment, up to as high as 1:4 (24, 26, 54). Globally, the World Health Organization (WHO) established the Commission on Social Connection to advance strategies and solutions that bring people together with the goal of improving health and wellbeing (55). In 2025, this Commission published a report recommending social prescribing as an approach to addressing the global crisis of loneliness and social isolation (56).

### 1.2 Arts-based social prescribing

Arts and cultural activities are commonly incorporated into social prescribing models due to the abundance of evidence suggesting a positive association between arts participation and health and wellbeing. Engagement with art has been shown to promote health and wellbeing (57, 58); improve cognitive and behavioral outcomes across the lifespan (51, 59–63); reduce risk for chronic health conditions (64–67); and decrease loneliness and isolation among older (68–70) and young adults (71, 72). In clinical settings, art therapy interventions have been used to complement behavioral health services (73). In community settings, arts activities provide spaces for social interaction and connection, offering an evidence-based option for supporting mental health that may be more accessible and less stigmatizing than psychotherapy, while avoiding concerns about side effects from psychiatric medication (23, 39, 51, 57, 59–61, 63, 66, 67, 70, 72, 74–93). The WHO has recognized the power of arts on health and has developed strategies for leveraging arts in the prevention and treatment of non-communicable diseases (94). Within care settings, arts-based social prescribing is an important strategy for addressing patients' whole health, in partnership with the community-based arts and culture sector (95).

While arts-based social prescribing models demonstrate positive outcomes for mental wellbeing, depression, and anxiety (51), evidence on how to effectively implement and scale these types of interventions in the US is emerging. Art Pharmacy, a US-based social prescribing program, is pioneering this approach with specific focus on integrating these services into U.S. health care (96).

### 1.3 About Art Pharmacy

Art Pharmacy (operated by SocialRx Inc., a private U.S.-based social prescribing organization) is an arts-based social prescribing program that helps address unmet mental health needs and supports social connection in the US through personalized arts and culture-based social prescriptions. The personalized social prescription is aimed at supporting individuals' mental health in an innovative way that aligns with their personal interests and supports achievement of their health goals. Art Pharmacy's care delivery model is informed by behavior change theory (97), behavioral activation for long-term habit forming and maintenance, with the goal of supporting sustained engagement in arts activities (83, 97–101). Art Pharmacy's model aligns with the “internationally accepted conceptual and operational definition of social prescribing” as described in the “Common Understanding of Social Prescribing Framework” put forth by Muhl et al. (40). Art Pharmacy prescribing partners across health care, community, and university systems identify and refer potential participants based on an indication of loneliness, social isolation, depression, or anxiety exhibited in an encounter or with existing screening tools in their practice. Art Pharmacy Care Navigators [link workers (42, 43)] receive referrals from prescribing partners and build relationships with participating individuals, or members, through phone, email, or text. They then match members with personalized community-based activities that reflect their health goals, interests, and needs; provide navigation and support services to attend these activities (including arranging transportation); follow up with regular communication to promote engagement; and keep the referring provider apprised of the member's progress. Care Navigators receive training in motivational interviewing and behavioral activation principles, mental health first aid, and evidence-based social prescribing practice. Care Navigators use Art Pharmacy's proprietary smart matching technology to tailor recommendations for arts activities. These activities can include workshops, classes, performances, and more across artistic disciplines (i.e., visual art, music, dance and movement, cultural experiences) and delivery modes (i.e., group or individual; participatory or receptive), provided by a diverse range of artists, institutions, and community-based organizations (102). Through Art Pharmacy, members have access to approximately one arts activity per month for 12 months to support regular and repeated engagement (89), promoting habit formation and health behavior change (103). Members also have the option to bring one companion to each activity to further support social connection.

Art Pharmacy works with public and private third-party payers across the U.S. health care ecosystem to cover the cost of social prescription services, eliminating or significantly reducing out-of-pocket expenses for members. Early implementation of the Art Pharmacy model was conducted in partnership with Medicaid managed care plans, Medicare provider groups, community mental health centers, cancer and palliative care providers, and colleges and universities, for people struggling with social isolation and loneliness, anxiety, and depression. These partner organizations were interested in scalable mental health support options that could complement existing behavioral health services and offer culturally responsive options. Art Pharmacy expands access to the health-promoting benefits of the community-based arts sector for people who may not otherwise be able to engage due to myriad structural barriers (104–106), including people from historically marginalized or underserved communities who are often not adequately served by the existing behavioral health workforce or service models (81, 107–109). Art Pharmacy provides a multi-level intervention that reduces transportation and financial barriers to engagement and supports equitable access to community-based arts participation for mental health.

As part of the model, Art Pharmacy regularly assesses mental health and wellbeing using validated scales, including the World Health Organization (WHO) five-item wellbeing scale (110–112). Assessments are administered upon enrollment in Art Pharmacy and following each arts activity in the social prescription. Collectively, these assessments inform both care delivery and evaluation of the model.

### 1.4 Evaluating mental health changes during Art Pharmacy participation

This research draws on early participant data from Art Pharmacy's social prescribing program. While Art Pharmacy was established in late 2022, data collection started in May 2023 for the WHO-5 Wellbeing Index. Secondary analysis of programmatic data and WHO-5 assessments was conducted to examine changes in mental health during Art Pharmacy participation. This research contributes to the growing evidence base examining associations between social prescribing and mental health and wellbeing, especially in the context of the US (113, 114).

## 2 Methods

The purpose of this study was to examine changes in mental health among Art Pharmacy members over time during participation in the social prescribing program. This secondary data analysis was approved by the Johns Hopkins University IRB (IRB00033793) as part of the primary author's doctoral studies.

The analysis included a sample of adult (aged 18 and over) participants who had completed the Art Pharmacy social prescription program by the summer of 2025 and for whom baseline and at least one follow-up assessment was available. We examined changes in WHO-5 wellbeing scores among 240 participants. Participants were assessed at baseline and at up to 14 follow-up time points over approximately 36 months, producing 780 total observations. Follow-up assessments ranged widely from 1 to 15 per participant (median = 2, IQR: 1–5), reflecting variation in participant engagement and program implementation timing. To address smaller sample sizes at later time points (fewer than 10 observations after Follow-up 9), the analysis was truncated at Follow-up 9, resulting in 761 observations from 240 participants and retaining 97.5% of total data. Data in this sample was collected between May 12, 2023, and July 11, 2025, from participants in California and Georgia.

The WHO-5 Wellbeing Index is a clinically validated 5-item self-report measure assessing psychological wellbeing and mental health over the past 2 weeks (111). Scores range from 0 to 100, with higher scores indicating better mental health. The commonly cited threshold for a clinically relevant change in wellbeing is 10 points. Scores of 50 or lower indicate possible depression, and scores of 28 or lower approximate a diagnosis for depression based on systematic literature reviews (110).

Descriptive statistics were produced for all participants in the dataset. Chi-squared tests, one-way analysis of variance (ANOVA), and two sample t-tests were used to examine selection and attrition bias. We used general estimating equations (GEE) with an exchangeable correlation structure to account for within-person correlation across repeated measures and examine participant-level changes across multiple time points. The models used a Gaussian family with an identity link function and robust standard errors, clustered on participant ID (115–120). We were not able to empirically assess the missing completely at random (MCAR) assumption as the possibility remains that missingness is related to unobserved values. To assess the potential impact of missing demographic data, we compared estimates from models fit in the full sample to those fit in the demographic-complete subsample; the consistency of results across these models suggests that missingness is unlikely to have substantially biased our findings. Four sequential models were tested: (1) unadjusted categorical time effects for each follow-up period with no covariates; (2) baseline-adjusted time effects to account for differences in participants' mental health at the start of the social prescription; (3) demographic-adjusted effects including gender (categorical female/male), age (continuous), Hispanic/Latino ethnicity (yes/no), and race (collapsed categories of White, Black/African American, Asian, Other/Multiracial/Prefer Not To Say); and (4) treatment-adjusted, adding in treatment duration in months to Model 3. In Models 3 and 4, the analytic sample decreased to 535 observations from 145 participants due to missing demographic data, representing a 39% reduction relative to Model 2. Model 2 was selected as the primary analysis because it retained most participants (99.6%), adjusted for individual baseline differences, and minimized selection bias from missing demographic data. Models 3 and 4 are reported as sensitivity analyses to examine demographic associations and treatment duration effects.

Missing data patterns were examined to assess attrition bias. Baseline WHO-5 scores were compared by gender and by demographic data completeness using two-sample t-tests. No significant differences in baseline wellbeing were observed between participants with complete vs. missing demographic data overall. However, differences in missingness by gender were observed, with males having higher rates of missing demographic data than females (45 vs. 28%).

All analyses were conducted using STATA 18.0. Statistical significance was set at α = 0.05 (two-tailed). Results are reported as coefficients with 95% confidence intervals and *p*-values.

## 3 Results

The analytic sample included 240 unique participants, with a total of 780 observations. After truncation at Follow-up 9, this was reduced to 761 observations. Participants completed an average of 3.25 assessments each (SD = 3.1, range = 1–15). In examining retention, 45% of participants completed only a baseline assessment, 16% completed baseline and at least one additional assessment, and 39% completed three or more assessments (labeled as “completers”). The average age of the full sample was 35 years old, which increased slightly in the completed demographic sub-sample. The sample was overwhelmingly female (77%) and almost half identified as Black or African American (45%). The average baseline WHO-5 score for the full sample was 52.2 (*SD* = 17.4), slightly above the threshold indicating possible depression (score < 50). Two sample *t*-tests revealed that females in the sample had significantly lower average baseline WHO-5 scores (*n* = 630, mean = 49.9, *SD* = 18.8) compared to males (*n* = 94, mean = 54.3, *SD* = 18.4; *p* = 0.036). See Table 1.

**Table 1:** Sample characteristics and demographics.

Characteristic	Full Sample	Model 1^a^	Model 2^b^	Model 3^c^	Model 4^d^
Sample Size					
Participants (n)	240	240	239	145^**^	145^**^
Observations (n)	780	761	760	535	535
Observations/Participant, mean (SD)	3.25 (3.1)	3.17 (3.1)	3.18 (3.1)	3.69 (3.2)^***^	3.69 (3.2)^***^
Demographics					
Age, mean (SD)	35.4 (19.9)	35.4 (19.9)	35.4 (19.9)	36.7 (20.6)	36.7 (20)
Female, n (%)	186 (77.5%)	186 (77.5%)	186 (77.8%)	125 (86.2%)	125 (86.2%)
Male, n (%)	33 (13.8%)	33 (13.8%)	33 (13.8%)	20 (13.8%)	20 (13.8%)
Hispanic/Latino, n (%)	–	–	–	12 (8.3%)	12 (8.3%)
Race, n (%)					
White	–	–	–	16 (11.3%)	16 (11.3%)
Black/African American	–	–	–	64 (45.1%)	64 (45.1%)
Asian	–	–	–	56 (39.4%)	56 (39.4%)
Other/multiracial/prefer not to answer	–	–	–	6 (4.2%)	6 (4.2%)
Complete demographics, n (%)	145 (60.4%)	145 (60.4%)	145 (60.4%)	145 (100%)	145 (100%)
Treatment duration					
Months, mean (SD)	–	–	–	–	8.6 (3.2)
Median [IQR]	–	–	–	–	9 [6–11]
Range	–	–	–	–	1–16
Baseline WHO-5					
Overall, mean (SD)	52.2 (17.4)	52.2 (17.4)	52.2 (17.4)	49.7 (18.2)^**^	49.7 (18.2)^**^
Female, mean (SD)	49.9 (18.8)	49.9 (18.8)	49.9 (18.8)	49.4 (18.4)	49.4 (18.4)
Male, mean (SD)	54.3 (18.4)	54.3 (18.4)	54.3 (18.4)	51.4 (16.5)	51.4 (16.5)
Male-Female difference	−4.4	−4.4	−4.4	−2.0	−2.0
p-value	0.036	0.036	0.036	0.645	0.645
Completion & attrition					
Completers (3 + observations), n (%)	93 (38.8%)	93 (38.8%)	93 (38.9%)	73 (50.3%)^*^	73 (50.3%)^*^
Average WHO-5 baseline: completers/non-completers	50.6/53.2	50.6/53.2	50.6/53.2	49.0/50.4	49.0/50.4
p-value	0.263	0.263	0.263	0.639	0.639
Average age: completers/non-completers	42.6/30.8	42.6/30.8	42.6/30.8	42.6/29.9	42.6/29.9
p-value	< 0.001	< 0.001	< 0.001	< 0.001	< 0.001

^*^p < 0.05,

^**^p < 0.01,

^***^p < 0.001 comparing Models 3 and 4 to full sample or between groups as indicated.

^a^Model 1: full Sample.

^b^Model 2: time + Baseline WHO-5 (1 participant who was missing baseline was excluded).

^c^Model 3: model 2 + Demographics (gender, age, ethnicity, race).

^d^Model 4: model 3 + Treatment duration in months.

All models were truncated at Follow-up 9 due to small sample size for subsequent time points. WHO-5 Scores range from 0 to 100, with higher scores indicating better wellbeing and scores below 50 indicating possible depression. Completers were defined as participants who had three or more assessments, including baseline. No treatment duration data were missing in Models 3 and 4.

### 3.1 Tests for attrition and selection bias

One-way ANOVA tests were performed to examine potential attrition bias based on the number of assessments completed during program participation and found no evidence, as participants who completed more assessments did not have significantly different baseline scores (*F* = 1.31, *p* = 0.203). A two-sample *t*-test was run to examine if completers had significantly higher baseline mental health scores than non-completers and found no evidence of selective attrition based on baseline WHO-5 scores [mean for completers = 50.6 (*SD* = 19.3); mean for non-completers = 53.2 (*SD* = 16.1); difference = −2.6, (*p* = 0.263)].

Demographic differences by completion were also examined. We found no evidence of differences in completers vs. non-completers by gender based on a chi-square test comparing male and female participants (χ^2^ = 0.162, *p* = 0.687). However, we did find significant differences in average age among completers vs. non-completers based on a two-sample *t*-test. Completers tended to be older (mean = 42.6 years, *SD* = 22.5), compared to non-completers (mean=30.8 years, *SD* = 16.6), with a mean difference of 11.8 years (*p* < 0.001). This finding likely reflects early implementation patterns of the Art Pharmacy program, which included a higher proportion of older adults referred from primary care and behavioral health providers. Although youth were also referred from community- and school-based mental health providers during the early implementation time, youth under 18 were not included in this analysis.

We examined demographic data completeness for selection bias. Because the data is derived from the beginning of program implementation, in which the full systems and structures for capturing programmatic and demographic data were not fully established, substantial missing demographic data was observed. Overall, 60% of the sample had complete demographic data for age, gender, race and ethnicity. Participants with complete demographic data had significantly lower baseline WHO-5 scores compared to those without (49.7 vs. 56.0 *p* = 0.0005), which has implications for Models 3 and 4.

**Figure 1:**
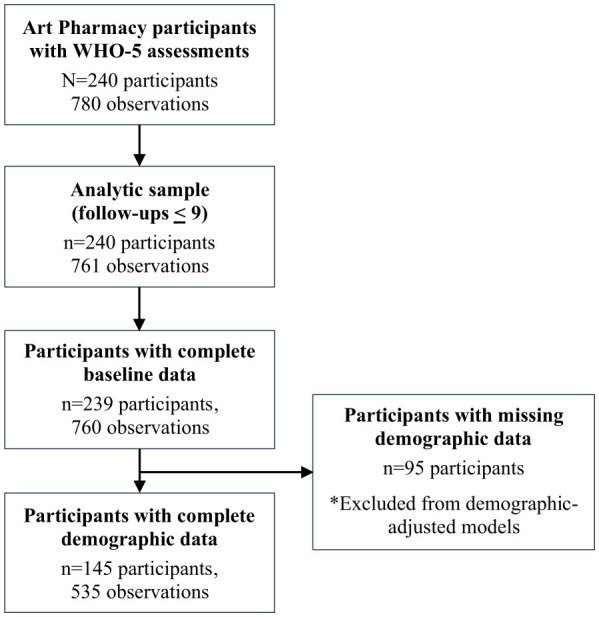
Participant flow and analytic sample selection.

Generally, attrition in assessment completion increased over time, with follow-ups 10 through 15 containing fewer than 10 participants each (16 total assessments). Overall, 93 participants (38.8%) were classified as completers (three or more assessments), while 147 participants (61.3%) completed two or fewer assessments. Figure 1 illustrates participant inclusion and the construction of the analytic sample used in the longitudinal analyses.

### 3.2 Mental health outcomes over time: general estimating equation (GEE) results

Our primary analysis focused on Model 2 (*n* = 239), which retained nearly the full analytic sample and adjusted for baseline differences in mental health and wellbeing (see Table 2). After controlling for baseline mental health scores in Model 2, the scale parameter decreased from 410.49 to 223.55, indicating that baseline mental health explained substantial variation in the outcome. Baseline WHO-5 scores were highly predictive of higher scores at follow-up (β = 0.76, *p* < 0.001). Participants demonstrated statistically significant improvements in mental health across all follow-up time points. Across all models, a pattern emerged of early improvements, with gains building over time to a peak at follow-ups six through eight, followed by sustained improvements through follow-up 9. Participants demonstrated immediate improvements at follow-up 1 (+5.5 points, 95% CI: 2.3–8.7, *p* = 0.0001), which increased to an additional 18–20 points at follow-ups 6 through 8, representing twice the meaningful improvement threshold of 10 points (all time points *p* < 0.0001). Based on the baseline standard deviation (*SD* = 17.4), the peak improvement of approximately 18–20 points corresponds to a large standardized effect size (Cohen's *d* = 1.03–1.15). At follow-up 9, mental health improvements remained (+13.2, 95% CI: 7.7–18.8, *p* < 0.0001).

**Table 2:** GEE models of WHO-5 mental health and wellbeing score increase trajectories^e^.

Variable	Model 1: unadjusted β (95% CI)	Model 2: baseline adjusted β (95% CI)	Model 3: demographics β (95% CI)	Model 4: treatment duration β (95% CI)
Time effects (ref: baseline)				
Follow-up 1	5.378 (2.2–8.6)^***^	5.519 (2.3–8.7)^***^	5.436 (1.2–9.7)^*^	5.430 (1.2–9.7)^*^
Follow-up 2	11.095 (7.5–14.7)^***^	11.153 (7.5–14.8)^***^	11.838 (7.1–16.6)^***^	11.828 (7.1–16.5)^***^
Follow-up 3	9.245 (5.0–13.5)^***^	9.101 (4.8–13.4)^***^	10.129 (4.7–15.5)^***^	10.120 (4.7–15.5)^***^
Follow-up 4	8.243 (4.6–11.9)^***^	7.924 (4.2–11.6)^***^	6.823 (2.5–11.1)^**^	6.808 (2.5–11.1)^**^
Follow-up 5	14.069 (9.0–19.1)^***^	13.763 (8.6–18.9)^***^	16.195 (10.1–22.2)^***^	16.181 (10.1–22.2)^***^
Follow-up 6	18.808 (13.8–23.8)^***^	18.583 (13.5–23.6)^***^	20.257 (14.7–25.8)^***^	20.245 (14.7–25.8)^***^
Follow-up 7	16.737 (11.5–22.0)^***^	16.510 (11.3–21.7)^***^	18.239 (12.6–23.9)^***^	18.226 (12.6–23.8)^***^
Follow-up 8	20.391 (14.1–26.7)^***^	20.124 (13.9–26.4)^***^	21.292 (14.5–28.1)^***^	21.275 (14.4–28.1)^***^
Follow-up 9	13.618 (8.0–19.3)^***^	13.244 (7.7–18.8)^***^	14.091 (8.0–20.1)^***^	14.068 (8.0–20.1)^***^
Baseline WHO-5	–	0.763 (0.67–86)^***^	0.694 (0.56–0.82)^***^	0.695 (0.57–0.82)^***^
Demographics				
Male	–	–	8.217 (2.8–13.7)^**^	8.215 (2.8–13.6)^**^
Age	–	–	−0.070 (−0.18–0.04)	−0.072 (−0.18–0.04)
Hispanic/latino	–	–	3.058 (−4.8–10.9)	2.959 (−5.1–11.0)
Race (collapsed)	–	–	0.103 (-2.5–2.7)	0.251 (−2.5–3.0)
Treatment months	–	–	–	0.153 (−0.44–0.75)
Constant	52.149 (49.9–54.4)^***^	12.379 (7.3–17.4)^***^	16.570 (5.9–27.3)^**^	14.945 (2.6–27.3)^**^
Model statistics				
Observations	761	760	535	535
Participants	240	239	145	145
Wald χ^2^	73.14	519.39	432.43	426.47
*p*-value	*p* < 0.001	*p* < 0.001	*p* < 0.001	*p* < 0.001
Scale parameter	410.49	226.55	242.62	242.16

^***^p < 0.001,

^**^p < 0.01,

^*^p < 0.05.

WHO-5 scores range from 0 to 100, with higher scores indicating better wellbeing. All models used general estimating equations with Gaussian family, identity link, and exchangeable correlation structure. Coefficients represent changes in WHO-5 scores relative to baseline. Model 2 added baseline WHO-5 as a covariate; Model 3 was restricted to participants with complete demographic data (n = 145, 60.2% of the Model 2 sample); Model 4 added treatment duration to Model 3 covariates. β= regression coefficient.

^e^Truncated at Follow-up 9.

In analyses restricted to participants with complete demographic data (Model 3; *n* = 145), the trajectory of improvement remained robust, with similar or slightly larger effect estimates (Follow-up 8, +21.3 points, *p* < 0.0001). Male gender appeared to be associated with higher WHO-5 scores (β = 8.2, 95% CI: 2.8–13.7, *p* = 0.003). This effect seems to have emerged during program participation, rather than reflecting pre-existing baseline differences (which were not significant in this subsample, despite being significant in the full sample). No other demographic variables showed significant associations (all *p* > 0.19). Adding treatment duration in months to Model 3 covariates for Model 4 did not improve model fit, with treatment duration showing no significant association with WHO-5 scores (β = 0.15, 95% CI: –.45–0.76, *p* = 0.617). All other effects remained unchanged.

## 4 Discussion

Findings from this analysis indicate that participants experienced statistically significant and sustained increases in self-reported wellbeing during participation in Art Pharmacy's social prescribing program. Improvements in WHO-5 scores were observed across follow-up assessments, with participants demonstrating similar positive trajectories of change regardless of baseline scores. The peak increase of 18-20 points represents double the established meaningful threshold of 10 points, indicating a significant and substantial improvement of participants' mental health during the social prescription. These trajectories remained stable and significant across models, demonstrating the strength of these patterns regardless of demographics or treatment duration. These findings provide preliminary observational evidence suggesting potential benefits of Art Pharmacy's arts-based social prescribing service on supporting mental health and reductions in depressive symptoms (as approximated with the WHO-5). These findings extend prior evidence demonstrating the benefits of social prescribing on mental health and wellbeing established in the field (51, 121) and provide novel, longitudinal evidence from a US arts-based social prescribing program. The use of a relatively large sample with multiple follow-up time points contributes to understanding about how arts-based social prescribing may be integrated into the US health care system as a complementary approach that may enhance mental health care alongside traditional clinical services.

Among participants with complete demographic data, we observed slightly stronger effects of program participation on WHO-5 scores. These participants started with significantly lower baseline mental health, demonstrating the strength of the effects of the program on people experiencing potential mental health concerns. In this analysis, male participants exhibited greater improvements in WHO-5 scores over time than female participants. This may partially reflect common gender differences in self-reported mental health, rather than differences in experiences of distress, as males tend to self-report lower mental health concerns but have higher rates of suicide (122). However, the benefits experienced by male participants may point to the potential for social prescribing as a strategy to address male mental health concerns and loneliness. Notably, no significant differences in mental health benefits experienced during the program were observed based on race, ethnicity, or age, suggesting similar patterns of improvement across population subgroups. These findings suggest that arts-based social prescribing may represent a broadly applicable community-based approach to supporting mental health across diverse populations. Further, the non-clinical community-based activities included in Art Pharmacy's social prescription program may help overcome access barriers to mental health supports, as these are readily available across most communities in the US, and in most cases, at lower cost than traditional behavioral health support options.

We found that longer treatment duration in terms of months does not predict better outcomes beyond the effects observed across follow-up time points. This finding suggests that sustained engagement in social prescribing activities, rather than cumulative time enrolled in the program, may be driving improvements in mental health. While participants experience mental health benefits immediately, sustained engagement across multiple activities (optimally more than six) during the social prescription appears to maximize mental health benefits. The trajectory-based improvement observed across follow-ups supports Art Pharmacy's theoretical basis of regular and ongoing engagement in arts-based social prescription activities. These findings are consistent with the behavior change and habit-forming framework of the model; supporting participant's ability to repeatedly engage in the arts is most effective at improving mental health. Further, the strength of the effects tied to follow-up assessments in this analysis is worth exploring in future research, as it may suggest that the structured post-activity survey and assessment process may itself be therapeutic; reflecting on social prescription program activities and mental health after participation may serve as an important reinforcing factor for the benefits of arts, cultural, and community engagement on mental health. Future research should incorporate more direct measures of participation intensity, such as attendance counts or number of activities completed, to better asses the dose-response relationship between arts engagement and mental health outcomes. While the sample reflects early program implementation patterns, the observed improvements in wellbeing provide preliminary evidence supporting further evaluation of arts-based social prescribing in more diverse populations and settings.

### 4.1 Limitations

This analysis has several limitations that should be considered when interpreting the findings. First, attrition across follow-up assessments was substantial, with 45% of participants completing only a baseline assessment. Although analyses did not identify evidence of selective attrition based on baseline WHO-5 scores, differential loss to follow-up may influence the observed results. Participants who remained engaged in the program and completed additional assessments may differ systematically from those who did not complete follow-up assessments, which could bias estimates of changes in wellbeing over time. To address sparse data resulting from additional attrition at later assessments, the analysis was truncated at Follow-up 9; however, differential attrition over time may still limit inferences about longer-term trajectories of wellbeing. Because follow-up participation varied and demographic data were incomplete, we cannot empirically verify the MCAR assumption. However, estimates were substantially consistent across models estimated in the full sample and the demographic-complete subsample, suggesting that missingness in demographic covariates is unlikely to have meaningfully biased our findings. Second, the demographic-adjusted models exhibited some evidence of selection bias, as participants with complete demographic data had lower baseline mental health scores than the full sample. As a result, these models were treated as sensitivity analyses. Third, the demographic composition of the sample may limit generalizability. Participants were predominantly female and relatively young, and the program was implemented in California and Georgia during the study period. These characteristics may not reflect the broader population of individuals who could participate in arts-based social prescribing programs. Future research should examine the effectiveness of similar models in more diverse geographic settings and among populations with different demographic and socioeconomic characteristics. Fourth, this study employed an observational design with no control group. Consequently, improvements in wellbeing observed during program participation may reflect, in part, unmeasured factors such as regression to the mean, concurrent supports, expectancy effects, or other unmeasured confounding factors. Ongoing research is examining the effectiveness of the Art Pharmacy social prescription program on subpopulations of interest including youth, older adults, and college students, using controlled study designs and accounting for treatment context. Fifth, outcomes were based on self-reported measures, which introduces the potential for favorability bias. Although validated instruments were used, changes in wellbeing may have been influenced by participants' awareness of program participation or recency bias related to experiencing arts-based social prescribing activities. Finally, this analysis examines early implementation of a single arts-based social prescribing program in the US. While the findings provide important evidence regarding Art Pharmacy and the potential of arts-based social prescribing in this context, they may not generalize to other programs, populations, or implementation models.

### 4.2 Strengths

A key strength of this analysis is the examination of mental health outcome trajectories across multiple time points. This longitudinal approach provides insights into patterns of change over time and informs understanding of how engagement in arts-based social prescribing may promote mental health. Methodologically, the use of generalized estimating equations allowed for appropriate modeling of repeated measures and within-person correlation, while retaining most available data. Sensitivity analysis further supported the robustness of the observed patterns. Importantly, we found no evidence of selective attrition based on baseline mental health, suggesting that improvements were observed across varying levels of baseline need.

Additionally, this study draws on one of the largest early datasets examining arts-based social prescribing program participants in the US, addressing a notable gap in the predominantly international evidence base about social prescribing outcomes. The positive improvements in wellbeing observed here are consistent with findings from social prescribing programs in other countries, supporting the relevance of this model in the US. These findings, which show statistically significant and meaningful increases in wellbeing associated with participation in an arts-based social prescribing program, may provide insight into program design and scale.

## Data Availability

The data analyzed in this study is subject to the following licenses/restrictions: The limited dataset used in this analysis was derived from programmatic data collected from participants in the the Art Pharmacy program for the purpose of program evaluation. The data was not collected or used with the intention of public dissemination and may not be shared publicly based on IRB approved protocols and data use agreements. It cannot be released or used by other researchers. Requests to access these datasets should be directed to Lucy Bailey, lrabino1@jhu.edu.
